# Cell replacement therapy with stem cells in multiple sclerosis, a systematic review

**DOI:** 10.1007/s13577-023-01006-1

**Published:** 2023-11-21

**Authors:** Maria Veatriki Christodoulou, Ermioni Petkou, Natalia Atzemoglou, Eleni Gkorla, Aikaterini Karamitrou, Yannis V. Simos, Stefanos Bellos, Chryssa Bekiari, Panos Kouklis, Spyridon Konitsiotis, Patra Vezyraki, Dimitrios Peschos, Konstantinos I. Tsamis

**Affiliations:** 1 Department of Physiology, Faculty of Medicine, School of Health Sciences, University of Ioannina, Ioannina, Greece; 2 Laboratory of Anatomy and Histology, School of Veterinary Medicine, Faculty of Health Sciences, Aristotle University of Thessaloniki, Thessaloniki, Greece; 3 Laboratory of Biology, Department of Medicine, University of Ioannina, Ioannina, Greece; 4 Department of Neurology, University Hospital of Ioannina, Ioannina, Greece

**Keywords:** Demyelination, Remyelination, Multiple sclerosis, Neural stem cells, Oligodendrocytes, Stem cell, Transplantation

## Abstract

Multiple sclerosis (MS) is a chronic inflammatory, autoimmune, and neurodegenerative disease of the central nervous system (CNS), characterized by demyelination and axonal loss. It is induced by attack of autoreactive lymphocytes on the myelin sheath and endogenous remyelination failure, eventually leading to accumulation of neurological disability. Disease-modifying agents can successfully address inflammatory relapses, but have low efficacy in progressive forms of MS, and cannot stop the progressive neurodegenerative process. Thus, the stem cell replacement therapy approach, which aims to overcome CNS cell loss and remyelination failure, is considered a promising alternative treatment. Although the mechanisms behind the beneficial effects of stem cell transplantation are not yet fully understood, neurotrophic support, immunomodulation, and cell replacement appear to play an important role, leading to a multifaceted fight against the pathology of the disease. The present systematic review is focusing on the efficacy of stem cells to migrate at the lesion sites of the CNS and develop functional oligodendrocytes remyelinating axons. While most studies confirm the improvement of neurological deficits after the administration of different stem cell types, many critical issues need to be clarified before they can be efficiently introduced into clinical practice.

## Introduction

Multiple sclerosis (MS) is a chronic autoimmune inflammatory disease of the central nervous system (CNS) primarily associated with demyelination of the neural axons but also leading to axonal degradation and neurodegeneration. As a consequence, neuronal impulses are not adequately transmitted and patients develop neurological symptoms. It is one of the main causes of disability in young adults and its incidence is increasing [1]. The pathogenesis of the disease is complex and has not yet been fully unraveled [2]. It is considered that the onset of the disease long precedes the first clinical symptoms. Existing immunomodulatory agents, despite being very efficient in reducing the rate of relapses, do not prevent progressive neurodegenerative processes, nor do they have any regenerative effect, while they may cause significant adverse effects [3].

A stem cell is an undifferentiated cell that can self-renew and differentiate into tissue-specific cell types. During the lifetime of an organism stem cells are able to act as repair cells, regenerating cells of organs. The behavior and properties of stem cells are regulated by their immediate environment, the niche [4]. Depending on their properties, stem cells can be divided into three main categories: pluripotent stem cells, totipotent stem cells, and multipotent stem cells. Pluripotent stem cells can differentiate into all tissue types except extra-embryonic tissues [5]. This category includes embryonic stem cells (ESCs) and induced pluripotent stem cells (iPSCs). ESCs are derived from the cells of the inner mass of the blastocyst during early embryogenesis and their differentiation in vitro is an important chapter in regenerative medicine [6]. iPSCs are generated in vitro from a patient’s own fully differentiated somatic cells by the process of cellular reprogramming [5], [7]. Totipotent stem cells include zygotes and blastomeres up to the eight-cell stage and have the ability to differentiate into all cell types needed to create a complete organism [8]. Finally, multipotent stem cells include stem cells of embryonic (fetal stem cells, FSCs) and adult tissues (adult stem cells, ASCs), and can differentiate into limited cell types of the tissue or the organ where they are found [5]. FSCs are mesenchymal-type cells that originate in the tissues of the fetus, can be found in the circulation during the first trimester of pregnancy and differentiate into bone, cartilage, haematopoietic cells, and oligodendrocytes [9]. ASCs are located in the niche of all body tissues, and their main function is to produce specialized cells for repair in case of damage, injury or disease. They include mesenchymal stromal cells (MSCs), hematopoietic stem cells (HSCs), stem cells from muscle tissue, and neural stem cells (NSCs) [10].

Due to their ability to self-renew and differentiate, stem cells have recently been proposed as a promising treatment for various degenerative and autoimmune disorders, including MS [11], [12]. The underlying mechanisms for the beneficial effects of administered stem cells include immunomodulation, transforming the central nervous system microenvironment from hostile to supportive and neurotrophic action, promoting the differentiation and regeneration of endogenous oligodendrocytes [13]. Despite all, their most interesting function is cell replacement, meaning their use as an exogenous source for the production of new oligodendrocytes that could possibly restore the damaged myelin sheaths. This review aims to present and clarify the role of stem cell replacement therapy in MS, to reveal the types of stem cells that perform cell replacement and their optimal route of administration.

## Materials and methods

A systematic literature search was conducted to identify eligible primary studies on stem cell replacement therapy in multiple sclerosis. Three medical and scientific databases (Medline, Embase, and Scopus) were searched, using the following search terms: “stem cell”, “cell replacement” and “multiple sclerosis”. No language or other restrictions were applied. The search spanned from inception of each electronic database to January 25th, 2022. Furthermore, the reference lists of published articles were searched manually, to ensure the comprehensiveness of the bibliography.

This effort resulted in 263 citations, from which relevant studies were selected for the review. Their potential relevance was examined and 129 studies were excluded as irrelevant. The full texts of the remaining 134 citations were assessed to select those primary studies that directly related to stem cell replacement therapy in multiple sclerosis. These criteria excluded 54 studies and left 80 in the review.

## Hematopoietic stem cells (HSCs)

HSCs constitute approximately 0.01% of the proliferating cells in the bone marrow and they can generate all hematopoietic cell lines, including erythrocytes, megakaryocytes and cells of the immune system [14]. Transplantation of HSCs was the first cell therapy to emerge for the treatment of MS and is currently the only clinically validated approach, having been introduced in the field of hematology, where it is mainly used for the treatment of malignancies [15]. In experiments performed in vivo in rodents, it was found that early treatment has substantial results in improving the clinical picture and prevention of relapses, while in contrast, in chronic stages of experimental allergic encephalomyelitis (EAE) the effect is negligible [16], [17]. The only comprehensive randomized clinical trial is the international autologous trial stem cell transplantation in MS, which compared mitoxantrone versus autologous HSC transplantation and included patients with aggressive relapsing–remitting MS (RRMS) and with secondary progressive MS (SPMS). Although a difference in EDSS scores was not observed, the results showed that autologous HSC was superior in terms of reducing MRI activity and recurrence rate [18]. The neurological disability observed in SPMS is mainly caused by neurodegenerative processes, due to axonal atrophy rather than inflammatory processes. As a result, the progressive phase may be curable by neither immunomodulatory agents nor autologous HSC [19], so there is a need to emphasize cell replacement with HSCs.

### Cell replacement with HSCs

Only two study was found to have encouraging results about the cell replacement that HSCs can accomplish in oligodendrocytes (Table 1). In the study of Goolsby et al., 2013, after autologous HSC and injection into the striatum and hippocampus, structural cell replacement was observed. CD34+ stem cells (HSCs) migrated long distances after injection into the shiverer brain, in many cases up to the contralateral hemisphere to the injection site. In some cases, the transplanted CD34+ cells selectively manifested oligodendrocyte MBP, in some cases, neuronal neurofilament H and NeuN, and in others astroglial GFAP. Finally, CD34+ cells from adult mouse bone marrow express classic MBP, extend oligodendroglial-like cell processes and ensheath axons in the brain and optic nerve [20]. Furthermore, in the clinical trial of Harris et al., 2020, HSCs were administered intravenously (IV) resulting in replacement of T cells. More than 90% of the pre-existing cerebrospinal fluid (CSF) repertoire in participants with active RRMS was removed following autologous HSC transplantation and replaced with clonotypes predominantly generated from engrafted autologous stem cells. The result was extensive removal of pre-existing T cell clonotypes in both CSF and CD4 T cells in peripheral blood. In addition, autologous HSCs replaced > 90% of the pre-existing T cell repertoire in CSF with new clones at month 24 after transplant [21].

**Table 1:** Study investigating cell replacement in MS with HSCs

Study	Type of cells	Experimental model	Administration route	Cell detection method	Result
Goolsby et al. 2013	Mouse HSCs CD34+	Adult shiverer mice	Injection into the striatum and hippocampus	Cell tracker orange (CTO) labeled HSCs	**Structural:** cell replacement CD34+ stem cells migrated long distances after injection into the shiverer brain, even to the contralateral hemisphere to the injection site CD34+ cells from adult mouse bone marrow expressed MBP, extend oligodendroglial-like cell processes and ensheath axons in brain and optic nerve In some cases, the transplanted CD34+ cells expressed neuronal neurofilament H and NeuN, and in others astroglial GFAP

## Mesenchymal stromal cells (MSCs)

Mesenchymal stromal cells (MSCs) are self-renewing cells with a role in supporting HSCs within the bone marrow vault and maintaining vascular and immune homeostasis, through their ability to selectively migrate to sites of tissue damage or inflammation (‘homing’). They have been extensively studied in multiple disease models as they are an easily accessible source of autologous or allogeneic somatic stem cells with the ability to differentiate in multiple directions [22, 23]. Moreover, they escape immunological surveillance and can be transplanted from an autologous, allogeneic source, even as a xenograft. It is noteworthy that their immunomodulatory, immunosuppressive, neurotrophic and repair functions may contribute to the treatment of MS [24].

The properties of MSCs that have been shown to be of potential therapeutic value for MS are as follows:

- Myelin repair: differentiation into cells of neuroendothelial origin and replacement in the injured CNS, stimulation of proliferation of endogenous CNS neural stem cells [25, 26], and guidance of their differentiation towards oligodendrocyte lineages [27].
- Suppression of inflammation and immunomodulation
- Neuroprotection through neuroprotective, antioxidant agents, promotion of CNS neurite outgrowth and remodeling.
- Reduced formation of gliotic scar: through their paracrine action, they can modify brain’s cellular microenvironment leading to a significant reduction of the lesion area [26]
- Promotion of angiogenesis, enhances tissue repair [28]
- Cell fusion: a mechanism of neuroprotection, whereby healthy nuclei or functional genes are introduced into damaged cells and help rescue them and restore function [29]
- Direct transfer of mitochondria to vulnerable cells via membrane fusion with subsequent phagocytosis [30]

### Cell replacement with MSCs

Several preclinical studies have been performed with administration of MSCs from various sources in animal models of MS, in which an improvement of the clinical picture and repair of the injured tissue has been observed [31], without clarifying whether the phenomenon is due to cellular replacement or paracrine and other actions of MSCs. There are on the other hand a number of studies that have proven cellular replacement with MSCs (Table 2).

**Table 2:** Studies investigating cell replacement in MS with MSCs

Study	Type of cells	Experimental model	Administration route	Cell detection method	Result
Croitoru-Lamoury et al. 2006	Human MSCs bone marrow derived	Twitcher model on mice	Intracerebral injections	GFP and CM-DiI labeled MSCs	**Structural:** The grafted MSCs retained rounded or flattened cellular morphologies with few arborizations. The MSCs integrated and remained around the site of injection but did not migrate extensively in the CNS parenchyma 1–14 days after transplantation Implanted MSCs expressed neuronal, astrocytic, and oligodendrocytic markers **Functional:** No change of clinical course. The twitcher animals transplanted with MSC did not display statistically significant improvement of the motor or behavioral deficits during the post-transplantation survival period compared with control animals
Jiang et al. 2017	Rat MSCs placental derived and embryonic MSCs (as control)	EAE on rats	ICV and ITH	GFP-labeled MSCs	**Structural:** Transplanted embryonic and placental MSCs migrated into CNS and differentiated into cells expressing neural and glial lineage markers Both embryonic and placental MSCs attenuated perivascular/parenchymal infiltration and reduced CNS inflammation. They inhibited demyelination (reduced the loss of MBP expression) and reduced neuronal necrosis and apoptosis, prevented axon loss, and alleviated reactive astrocyte proliferation and reactive gliosis **Functional:** Both embryonic and placental MSCs reversed electrophysiological dysfunction, postponed the onset of motor symptoms and reduced disease severity
Akiyama et al. 2002	Mice MSCs bone marrow derived	EB-X model on rats	Direct injection into the demyelinated spinal cord	GFP-labeled MSCs	**Structural:** Extensive remyelination at 3 weeks. MSCs differentiated primarily into myelinating cells when injected into a demyelinated spinal cord lesion Many remyelinated axons expressed morphological features of peripheral myelin Colocalization was observed for MPB (myelin-specific protein) and P0 (peripheral myelin specific) with transplanted GFP-expressing stromal cells **Functional:** Remyelinated axons showed improved conduction velocity
Akiyama et al. 2002	Rat MSCs bone marrow derived, Schwann cells, olfactory ensheathing cells, residual cell fraction	EB-X model on rats	IV	LacZ gene transfection of MSCs and detection of β-galactosidase reaction products Fluorescent cell marker (PKH26)	**Structural:** Remyelination only after the IV delivery of the bone marrow-derived MSCs and not the other cell injections About 9% of myelin-forming cells in the lesion showed β-galactosidase reaction products Fluorescence was only detected in the lesion zone, leading to the assumption that the blood–brain barrier was compromised in the lesion zone and delivered cells entered **Functional:** A subpopulation of remyelinated axons in rats injected with bone marrow-derived MSCs showed partial recovery of electrophysiological function with increased conduction velocity
Kassis et al. 2008	Mice MSCs bone marrow derived	Chronic EAE on mice	ICV and IV	GFP-labeled MSCs	**Structural:** MSCs from both administration routes were attracted to the areas of CNS inflammation and showed morphological and immunohistological features of neuronal lineage cells Reduction in CNS inflammation and significant protection of the axons was observed, especially following intraventricular administration After IV administration, systemic immunomodulatory effects were observed with downregulation of lymphocytes **Functional:** The clinical course of chronic EAE was ameliorated in MSCs-treated animals (reduced mortality and clinical scores) compared to the control group
Zhang et al. 2006	Human MSCs bone marrow derived	EAE on mice	IV	Immunohistochemistry for MAB1281	**Structural:** Reduced axonal loss and increased axonal density was observed MSCs were present in the EAE brain from week 1 to 45, but no significant cell replacement was evident, since less than 5% of MAB1281+ cells colocalized with a marker of oligodendrocyte progenitor cells and nearly 10% colocalized with a marker of astrocytes and a marker of neurons **Functional:** MSCs reduced mortality, disease severity and number of relapses in EAE mice
El-Akabawy et al. 2015	Mice MSCs bone marrow derived	Non-immune cuprizone model on mice	IV	PKH-labeled MSCs	**Structural:** MSCs migrated, engrafted, reduced demyelination, and enhanced remyelination However, the detected remyelination was not graft-derived as no differentiation of the MSCs towards the oligodendroglial phenotype was detected. Few transplanted cells differentiated into the astroglial phenotype (co-expressed GFAP) but no sign of oligodendroglial differentiation was detected (no transplanted cells expressed CNPase: mature oligodendrocyte marker) Enhancement of endogenous repair and induction of oligo/neuroprotection were mentioned as possible mechanisms of action
Zhang et al. 2005	Human MSCs bone marrow derived	EAE on mice	IV	Mitotic labeling with BrdU and immunohistochemistry for MAB1281	**Structural:** Reduction of inflammatory infiltrates and demyelination in the CNS was observed MAB1281+ cells were located in the white matter of the spinal cord, corpus callosum, and striatum and entered the demyelination areas Less than 5% of MAB1281+ cells colocalized with a marker for oligodendrocyte progenitor cells, so no significant cell replacement took place Stimulation of endogenous oligodendrogenesis was observed and increased expression of BDNF **Functional:** MSCs treatment improved neurological functional recovery with significant decreases of the clinical scores
Gerdoni et al. 2007	Mice MSCs bone marrow derived	EAE on mice	IV	GFP-labeled MSCs	**Structural:** Reduced demyelination, inflammation, and axonal loss was observed MSCs entered the CNS but did not transdifferentiate into neural cells (no clear evidence of colocalization of neural markers and GFP) Interference with the pathogenic autoimmune response was mentioned as possible mechanism of action **Functional:** MSCs ameliorated the severity of the disease on EAE mice with fewer relapses compared with control
Gordon et al. 2008	Human MSCs bone marrow derived	EAE on mice	Intraperitoneal injection	GFP-labeled MSCs Anti-human nuclear antigen-specific Ab-labeled MSCs	**Structural: **NO cell replacement Profoundly little CNS infiltration in demyelinated lesions and normal CNS was observed Peripheral or systemic immune effect was mentioned as possible mechanism of action **Functional:** Substantial amelioration of clinical disease in MSCs-treated mice
Bai et al. 2009	Human MSCs bone marrow derived	EAE on mice	IV	CMFDA or CFSE-labeled MSCs	**Structural:** Reduction of inflammatory cells and demyelination was observed MSCs were found in regions of demyelination in EAE animals enhancing endogenous repair in part by stimulating oligodendrogenesis and reducing inflammatory cells No evidence of MSCs adopting a neural fate was detected based on expression of neuronal or glial antigens **Functional:** MSCs ameliorate chronic and relapsing–remitting EAE, improving clinical scores
Hunt et al. 2008	Rat MSCs bone marrow derived	EB-X model on rats	Intralesional injection	GFP-labeled MSCs	**Structural:** Transplanted MSCs did not remyelinate the axons in the lesion sites, they expressed mesenchymal markers and no neuronal or glial markers MSCs migrated into areas of normal tissue, deposited collagen and were associated with axonal damage
Grigoriadis et al. 2011	Autologous MSCs bone marrow derived	EAE (mild and severe) on mice	ICV	GFP-labeled MSCs	**Structural:** In the mild EAE model, MSCs exerted significant anti-inflammatory effect on spinal cord with concomitant reduced axonopathy In the severe EAE model, MSCs migrated into the brain parenchyma and formed cellular masses characterized by focal inflammation, demyelination, axonal loss and increased collagen-fibronectin deposition (via both paracrine and autocrine manner) **Functional:** MSCs significantly ameliorated mild though not severe EAE

#### EAE model

Nine of the experimental MSCs transplantation studies in the Table 2 were performed in rodent models with EAE and resulted in improved neurological function. Five of the studies used rodent MSCs [32–36], derived from bone marrow and placenta, while the other four used human MSCs isolated from bone marrow [36–39]. In two of the studies using rodent MSCs, cell replacement of various degrees was observed [35, 36], whereas no cell replacement was observed after administration of human MSCs. The therapeutic effect of intracerebroventricularly (ICV) and intrathecally (ITH) administered rat MSCs was partially attributed to cell replacement, as they trans-differentiated into cells expressing markers of neuronal and neuroglial phenotype, such as NF-200, Olig1, MBP, and GFAP [35]. The anti-inflammatory activity of MSCs also played an important role, as they attenuated perivascular and parenchymal infiltration, suppressed proinflammatory factors and increased the expression of anti-inflammatory cytokines. Similarly, in the study by Kassis et al*.* [36], both IV and ICV administered murine MSCs derived from bone marrow, have exhibited cell replacement, with MSCs being attracted to sites of CNS inflammation and displaying morphological and immunohistological characteristics of neuronal-directed cells, expressing beta-tubulin type III, GFAP, and galactosebroside. In addition, a reduction in CNS inflammation and significant protection of axons was observed, particularly after ICV injection, suggesting a possible local in situ immunomodulatory effect of MSCs. During IV administration, systemic immunomodulatory effects also played an important role through a reduction in lymphocyte proliferation.

In contrast, in the study by Grigoriadis et al*.* [33], where autologous MSCs from bone marrow were administered ICV to mice with EAE, cells displayed GFAP and NG2 phenotypes, but without sufficient morphological integration within the tissue. In the mild EAE model, MSCs exerted anti-inflammatory activity in the spinal cord and reduced axonopathy. However, in the severe form of EAE, significant adverse effects were observed, with the formation of cell masses in the brain parenchyma, focal inflammation, demyelination, axonal loss and increased collagen and fibronectin deposition. In the experiment of Gerdoni et al*.* [32], IV administered mouse MSCs derived from bone marrow entered the CNS but did not trans-differentiated into cells of neural origin. However, they led to a reduction in demyelination and inflammation by interfering with the pathogenic autoimmune response, inhibiting the pathogenic T and B cell response and the proliferation and production of inflammatory cytokines of brain-derived T cells. In the experiments of Zhang et al*.* [37, 39] and Bai et al*.* [27], human bone marrow derived MSCs were injected IV into mice with EAE and entered the demyelination areas of the CNS. However, either no colocalization was observed, or only a small number of MSCs colocalized with neuroglial cell markers. Even if the colocalization of these markers is due to actual trans-differentiation of a small proportion of MSCs into oligodendrocyte progenitor cells, their therapeutic effect cannot be attributed to this. Instead, enhancement of endogenous repair by stimulation of oligodendrogenesis was found, as well as anti-inflammatory activity of MSCs, with a decrease in inflammatory TH1 and TH17 cells, an increase in anti-inflammatory TH2, and expression of neurotrophic factors, such as brain-derived neurotrophic factor (BDNF) and neural growth factor (NGF). Nor in the study by Gordon et al*.* [38], where human MSCs were administered intraperitoneally to EAE mice, did cell replacement take place, as very little infiltration by MSCs was observed in the CNS and the demyelinated lesions. It is, thus, speculated that the beneficial effect is due to a peripheral or systemic immune effect.

In the experiment of Harris et al*.* [40], bone marrow-derived mouse MSCs were in vitro differentiated into neural progenitor cells, in order to have a greater neurogenic potential. After intracranial injection in mice with EAE, a reduction in demyelination and a detection of the stem cells in inflammatory foci was observed. However, despite the in vitro neural differentiation potential of these cells, no evidence of in vivo trans-differentiation was detected. The transplanted cells led to reduced T cell infiltration, indicating an anti-inflammatory mechanism of action and enhanced endogenous repair, with the detection of increased numbers of endogenous NPCs. Clinically, improvement in neurological function was found with multiple intradural injections, while a single injection did not affect disease scores.

In experiments involving the transplantation of MSCs into EAE models, diverse outcomes were observed. This variability reflects the differences in the studies’ design mainly regarding the source of the MSCs and the site of transplantation. It could also stem from other factors, including the microenvironment encountered by MSCs during homing. This environment may either enable them to eventually replace cells or hinder their functionality. The causes of this variability could be an area of further research and may reveal new roles for MSCs. Undoubtedly, this phenomenon underscores the challenges associated with cell therapy using MSCs.

#### Chemical demyelination

Three studies used rats with focal demyelination in the spinal cord after ethidium bromide injections followed by irradiation (EB-X model), to exclude the possibility of endogenous remyelination by oligodendrocytes or Schwann cells for at least 6–8 weeks [41–43]. MSCs from rodent bone marrow were administered by direct injection into the lesion or IV. In the experiment of Akiyama et al*.* [42], GFP-labeled mouse MSCs were administered by direct microinjection into the EB-X model. Upon electron microscopy of the lesion at 3 weeks, relatively extensive remyelination was observed, which was almost complete near the center of the lesion and partial at the lateral borders. In the absence of endogenous repair and in the presence of intense GFP fluorescence, it was concluded that the injected MSCs differentiated mainly into myelinating cells, thus cell replacement occurred. Furthermore, the remyelinated axons were also shown to be functional, as they showed improved conduction velocity. Similarly, in another study by Akiyama et al*.* [41], rat MSCs, contaminated with the LacZ gene, were administered, this time IV, to rats with EB-X focal demyelination, leading to remyelination. Approximately, 9% of the cells that formed myelin in the lesion showed β-galactosidase reaction products, but the majority of myelinating cells did not. From this, it is concluded that cell replacement occurs to a small extent, but that some endogenous repair mechanism, probably enhanced by MSCs, is responsible for most of the induced remyelination. However, given issues related to the low efficacy of LacZ contamination and the possibility of gene inactivation as cells progress to a myelinating phenotype, we cannot discern whether the remyelination was caused by the injected cells or whether the injection procedure facilitated an endogenous repair mechanism. Interestingly, in the latter two studies, remyelinated axons showed, on electron microscopy, morphological features similar to either central myelination from oligodendrocytes or peripheral myelination from Schwann cells, with large nuclei and an enveloping basement membrane. In addition, colocalization of both MPB, a myelin-specific protein for both central and peripheral myelin, and P0, specific for peripheral myelin, was observed with transplanted stromal cells. Functionally, partial recovery of electrophysiological function was found, with improved axon conduction velocity.

Contrary, in a study conducted by Hunt et al*.* [43], the administration of MSCs not only did not lead to remyelination and cell replacement, but instead had deleterious consequences, as they migrated to areas of normal tissue where they deposited collagen and caused axonal damage. The study by El-Akabawy and Rashed [44], used mice with cuprizone-induced non-immune demyelination as an experimental model, which were injected IV with MSCs from mouse bone marrow. Cuprizone induces oligodendrocyte apoptosis and subsequent demyelination. MSCs migrated, integrated and led to both a reduction in demyelination and an enhancement of remyelination. However, the authors postulated that remyelination was not induced by the administered cells, as no differentiation of MSCs towards the oligodendroglial phenotype was detected (absence of CNPase expression). The putative mechanisms of action identified were the direct enhancement of endogenous repair and the induction of oligo/neuroprotection, via reduction of the neuroglial response (astroglia and microglia) to cuprizone and reduction of oligodendrocyte apoptosis.

#### Myelin twitcher mutant

In the experiment of Croitoru-Lamoury et al*.* [45], human bone marrow MSCs were administered via intracerebral injections into twitcher mice. In 28 of 40 animals, MSCs were successfully integrated and maintained rounded or flattened cellular morphologies with few dendritic appendages, but did not migrate extensively into the CNS until day 14 post-transplant. Differentiation of MSCs into CNS cells was observed, as they were found by immunostaining to express the neuron-specific protein MAP2, the astrocytic protein GFAP, and oligodendrocyte proteins, MBP and CNPase. However, functionally, no change in clinical progression was found.

## Neural stem cells (NSCs)

During the last decade, increasing interest has been focused on the use of neural stem cells (NSCs) to promote remyelination. In the adult CNS, tissue-specific stem sheaths, such as the subventricular zone (SVZ) of the lateral ventricles and the subgranular zone (SGZ) of the dentate gyrus of the hippocampus, contain the majority of endogenous NSCs with the ability to self-renew and differentiate into functional neurons and glia [46].

Pluripotent NSCs have also been isolated from the subcortical white matter of the adult human brain [47]. MS has been shown to significantly affect the proliferation of the endogenous NSCs and this effect has been correlated with clinical and histopathological findings in animal models of the disease [48]. NSCs in the adult mammalian brain produce rapidly dividing neural progenitor cells (NPCs) to generate neurons, astrocytes, and oligodendrocytes and contribute functionally to post-injury recovery processes [49]. For example, SVZ neuroblasts of adult mice can be directed mainly toward oligodendrocyte differentiation during lysolecithin-induced demyelination of the corpus callosum [50].

Apart from endogenous NSCs, transplanted NSCs and NPCs, which possess a high myelinogenic potential, provide encouraging results in animal models of MS. Exogenously administered NSCs and NPCs exert a beneficial effect on demyelination through cell replacement and immunomodulation, or by providing nutritional support, neuroprotection, and stimulating endogenous remyelination. For NSCs therapy in MS to be successful, the cells must possess sufficient plasticity to integrate and survive in the unfavorable inflammatory environment of the CNS, excellent migratory capacity to reach multiple lesion sites, and the ability to contribute to remyelination [13].

Several studies report that transplanted NSCs reduce the clinical and inflammatory signs of EAE, although they remain in the perivascular region without migrating to the site of injury [51–53] and suggest that NSCs exert beneficial effects not through cell replacement but through other mechanisms, such as immune regulation. In particular, transplantation of NSCs within brain ventricles reduces perivascular infiltrates, CD3+ T cells, and ICAM-1 and LFA-1 expression and increases T-regulatory cells in the brain and spinal cord [52, 53]. Adult NSCs overexpressing IL-10 significantly suppress CD45+ cells, CD4+ T cells, CD68+ macrophages/microglia, and CD8+ T cells in the spinal cord. They also inhibit the production of the inflammatory cytokines IFNγ and IL-17 and induce apoptosis of cerebellar T cells [54]. IV administration of NSCs reduces the number of CD3+ T cells and Mac3+ macrophages infiltrating the spinal cord [55], whereas subcutaneous injection of human NSCs inhibits T executive cell generation, dendritic cell differentiation and maturation, and cytokine production [56].

The nutritional and neuroprotective effects of NSCs are exerted through the delivery of neurotrophins, growth factors, stem cell developmental regulators and immunomodulatory molecules, all of which serve to regulate the microenvironment [57]. NSCs IV injected in an animal model of EAE secrete PDGF-α and FGF2, causing stimulation of proliferation and differentiation of oligodendrocyte progenitor cells (OPCs), and consequently, enhancing remyelination [58]. In EAE mice that were subcutaneously injected with NPCs before disease onset, NPCs accumulated in lymph nodes and inhibited myeloid dendritic cells [56]. Moreover, in chronic Alzheimer’s disease (AD), SVZ-derived congenic NSCs promote neuroprotection through the secretion of immunomodulatory molecules and neurotrophic factors [59].

Transplanted NPCs can stimulate endogenous remyelination by inducing the proliferation and terminal differentiation of endogenous OPCs. NPCs transplanted into the lateral ventricles of mice with cuprizone-induced demyelination exerted a trophic effect on endogenous OPCs, and the achieved remyelination in the corpus callosum was attributed exclusively to endogenous OPCs [58]. In another study, intradural injection of MSC-NPCs at the onset of the chronic phase of EAE in mice increased the number of endogenous OPCs and accelerated remyelination [34].

### Cell replacement with NSCs

The intrinsic ability of NSCs to differentiate into oligodendroglial lineage cells has fostered hope for their application as clinical therapies in MS. Many preclinical studies have been conducted in a variety of both rodents and non-human primates to investigate the therapeutic potential of transplanting NSCs or OPCs in MS. The current perspective on the predominant mechanisms underlying the beneficial effect of transplanted NSCs involves diverse graft-host interactions, not focusing on the replacement of damaged tissue [57, 60]. However, there is a number of preclinical studies in animal models of MS that have used NSCs or OPCs investigating their potential for cell replacement (Tables 3 and 4). These cells are generated in specific stem cell-containing regions of the CNS, from where they migrate extensively into axonal pathways that myelinate. They persist into adulthood and are the cells responsible for remyelination [61].

**Table 3:** Studies investigating cell replacement in MS with NSCs

Study	Type of cells	Experimental model	Route of Administration	Detection method	Result
Windrem et al. 2020	Human GPCs fetal brain derived	1. Shiverer mice 2. Cuprizone demyelination mice	Subcortical injection	Immunostaining for human nuclear antigen	**Structural**: Cell replacement: transplanted human GPCs dispersed broadly throughout the adult CNS, differentiated as oligodendroglia and broadly remyelinated demyelinated axons **Functiona**l: Improved host callosal conduction, motor function, and ambulation
Buchet et al. 2011	Human NPCs from two independent fetuses	1. Lysolecithin demyelination mice 2. Shiverer mice	Injection at the site of demyelination	GFP-labeled NPCs	**Structural**: Cell replacement: human NPCs survived, extensively migrated and gave rise to differentiated oligodendrocytes that successfully remyelinated the spinal cord **Functional**: Functionality of donor-derived myelin was assessed through clustering of Caspr at the paranodes and of Na_v_ channels at the nodes of Ranvier
Ben-Hur et al. 2003	New-born rat neural precursor cells: PSA − , NCAM+, nestin+, NG2 −	EAE rats	ICV or ITH	Nuclear fluorescent dye Hoechst or incubation with BrdU	**Structural**: Cells migrated exclusively into inflamed white matter but not into intact adjacent gray matter regions After 2 weeks, many transplanted cells had migrated into distant white matter tracts and acquired specific markers of the astroglial (GFAP) and oligodendroglial (NG2, GalC) lineages Inflammatory process may attracted targeted migration of remyelinating cells
Uchida et al. 2012	Human NSCs: CD133, Sox2, and nestin (+), O4 (-)	Shiverer mice	Injection into 3 distinct CNS sites	Human-specific nuclear and cytoplasmic markers (SC101, SC121)	**Structural**: Cell replacement: diffuse migration, engraftment, and differentiation into oligodendrocytes that generated compact myelin with normalized nodal organization, ultrastructure, and axon conduction velocities Human NSCs myelination resulted in fractional anisotropy changes detected by magnetic resonance imaging
Pluchino et al. 2003	Adult neural precursor cells (neurospheres)	EAE mice (chronic, relapsing model)	IV or ICV	Expression of b-galactosidase gene (LacZ)	**Structural**: Cell replacement: both IV and ICV significant numbers of donor cells entered demyelinating areas and differentiated into myelin-forming cells that actively remyelinated axons Stimulation of endogenous remyelination: marked increase of oligodendrocyte progenitors in demyelinated areas, with up to 20% of them being of donor origin. The remainder were of endogenous origin Significant reduction of reactive astrogliosis, decreased demyelination and axonal loss **Functional:** The functional impairment was almost abolished in transplanted mice, both clinically and neurophysiologically with initiation of recovery 10 days after IV injections and 15 days after ICV transplantation
McIntyre et al. 2020	Mouse and human NSCs	EAE mice (chronic phase)	Intraspinal	GFP-labeled NSCs	**Structural**: Mouse NSCs engrafted, migrated extensively from the transplant site and remyelinated. Reduction in demyelination is likely due to cell replacement, with little impact on immune cells Human NSCs promoted remyelination, regulatory T cell expansion and decrease in CD4+ T cells **Functional** No significant clinical response
Carbajal et al. 2010	NSCs	JHMV-infected mice (viral induced demyelination immune mediated)	ICV	GFP-labeled NSCs	**Structural**: Cell replacement: migration, proliferation, and differentiation of the cells into OPCs and mature oligodendrocytes that is associated with increased axonal remyelination
Greenberg et al. 2014	Mouse NPCs	JHMV-infected mice	Intraspinally at thoracic vertebra	GFP-labeled NPCs	**Structural**: Cell replacement: NPCs preferentially accumulated within areas of axonal damage and differentiated into myelinating cells that initiated direct contact with axons and expressed myelin genes, initiating remyelination
Akiyama et al. 2001	Human NPCs derived from the adult human brain	EB-X model on rats	Direct injection into the central region of the lesion	Expression of b-galactosidase gene (LacZ)	**Structural**: Cell replacement: NPCs elicited extensive remyelination with a peripheral myelin pattern like Schwann cell myelination, with large cytoplasmic and nuclear regions, a basement membrane, and immunoreactivity for P0 (peripheral myelin-specific protein) **Functional:** Impulse conduction at near normal conduction velocities in the remyelinated axons **Conclusion**: The CNS-derived NPCs differentiated in vivo into a cell with peripheral Schwann cell characteristics which formed functional myelin
Sher et al. 2012	Mouse NSCs and Olig2-NSCs (transient overexpression of Olig2)	EAE mice (chronic relapsing)	ICV	Luciferase/GFP-labeled NSCs	**Structural:** Cell replacement: NSCs and Olig2-NSCs directly migrated toward active lesions. The majority of Olig2-NSCs, in contrast to NSCs, differentiated into OPCs (NG2+). Survival of Olig2-NSCs was significantly higher than that of NSCs, which remained undifferentiated (Nestin+) **Functional**: Both Olig2-NSCs and NSCs significantly reduced the clinical signs of acute and relapsing disease and, in case of Olig2-NSCs, even completely abrogated relapsing disease when administered early after onset of acute disease
Copray et al. 2006	Mouse NSCs from the embryonic brain transfected with the Olig2 gene	Cuprizone demyelination of the corpus callosum in mice	Stereotactical implantation in the striatum	MiniRuby-labeled NSCs	**Structural:** Oligodendrocyte differentiation induction in vitro: overexpression of Olig2 induced the development of fully mature oligodendrocytes expressing the TF Nkx2.2 and all major myelin-specific proteins Cell replacement: after transplantation: Olig2-transfected NSCs, in contrast to nontransfected NSCs, developed into actively remyelinating oligodendrocytes that contributed to the remyelination of the denuded axons in the corpus callosum
Whitman et al. 2012	NPCs Olig1-/- and Olig1+/+	JHMV-infected mice	Direct implantation	GFP-labeled NPCs	**Structural:** Similar cell survival, proliferation, and selective migration to demyelination areas for NPCs Olig1-/- and Olig1+/+ Only recipients of wild-type (Olig1+/+) NPCs exhibited extensive remyelination compared to recipients of Olig1-/- NPCs (not able to remyelinate demyelinated axons) Cell replacement: the demonstration of transplanted NPCs derived myelin wraps surrounding axons provided compelling evidence that transplanted cells directly remyelinated axons Olig1+/+NPCs preferentially differentiated into NG2+ OPCs and formed processes expressing myelin basic protein that encircled axons Olig1-/- NPCs differentiated into GFAP+ cells of the astrocyte lineage **Conclusion:** Olig1 function is required for remyelination potential of transplanted NPCs
Booma et al. 1999	Mouse NSCs	Shiverer mice	ICV	Expression of b-galactosidase gene (LacZ)	**Structural:** Cell replacement: NSCs migrated, engrafted extensively within the brain parenchyma, and differentiated into oligodendrocytes, expressing MBP and therefore producing compact myelin that enwrapped host axons **Functional:** Extensive amelioration of symptoms: 60% of tested transplanted mutants evinced behavior that approximated normal
Vitry et al. 2001	Mouse 1. PSA-NCAM (+) NSCs 2. GD3(+) OPCs	Shiverer mice	Periventricular transplantation	Hoechst-labeling of spheres	**Structural**: Cell replacement: both transplanted cells’ populations differentiated into oligodendrocytes with similar myelination potential, but PSA-NCAM NSCs migrated more efficiently than GD31 OPCs. Also, PSA-NCAM NSCs cells generated GFAP(+) astrocytes and NeuN(+) neurons, depending on their site of insertion
Keirstead et al. 1999	Rat PSA-NCAM+ NSCs	EB-X model in rats	Direct injection into the lesion	Y chromosome in situ hybridization On female rats that had been grafted with male NSCs	**Structural:** Cell replacement: PSA-NCAM+ NSCs generated oligodendrocytes, astrocytes, and Schwann cells in vivo when confronted with demyelinated axons in a glia-free area. PSA-NCAM+ NSCs remyelinated the lesion very efficiently and, surprisingly, remyelination was carried out by both oligodendrocytes (81%) and Schwann cells (~ 19%)
Mothe et al. 2008	NPCs from the PVZ of adult rat spinal cord	1. EB-X model in mice 2. Shiverer mice	Injection into the spinal cord	GFP-labeled NSCs/NPCs	**Structural:** Cell replacement: in the EB-X model the transplanted cells primarily differentiated along an oligodendrocyte lineage but only some of the oligodendrocytic progeny remyelinated host axons, expressing MBP NPCs also differentiated into myelinating cells with Schwann-like features In the shiverer mice: the majority of the NPCs expressed an oligodendrocytic phenotype and robustly myelinated the dysmyelinated CNS axons forming compact myelin, and none had Schwann cell-like features **Conclusion**: PCs have the inherent plasticity to differentiate into oligodendrocytes or Schwann-like cells depending on the host environment, and both cell types are capable of myelinating axons
Giannakopoulou et al. 2011	Mice NPCs from cerebral hemispheres	EAE mice (acute and chronic phases)	ICV	GFP-labeled NPCs	**Structural:** Cell replacement at chronic phase: In vivo: acute phase of EAE: only a small fraction of transplanted NPCs succeed to differentiate At chronic EAE: most of them followed a differentiation process to glial cell lineage along white matter tracts. However, this differentiation was not fully completed, since 8 months after their transplantation a number of NPCs remained as pre-oligodendrocytes In vitro: glial differentiation of NPCs was inhibited or promoted following their treatment with TNFa (proinflammatory cytokine) or TGFb (anti-inflammatory cytokine) respectively **Functional**: Attenuation of severity of clinical disease (the most significant attenuation of the clinical paralytic signs was observed at the chronic phase) **Conclusion**: Inflammation triggers migration whereas the anti-inflammatory component is a prerequisite for NPCs to follow glial differentiation thereby providing myelinating oligodendrocytes
Windrem et al. 2002	Adult human subcortical white matter NSCs A2B5+ progenitor	Lysolecithin-demyelinated rat brain (in the corpus callosum)	Injection into the site of lysolecithin injection	Lipophilic dye tagging or anti-human nuclear antibody immunostaining or BrdU labeled or GFP tagging	**Structural:** Cell replacement: implanted NSCs migrated rapidly and extensively through regions of experimental demyelination (but not when implanted into normal brain) and differentiated as myelinating MBP+ oligodendrocytes that projected lamellipodia At the lesion borders, a preponderance of GFAP+ GFP-tagged cells was typically noted, indicating the astrocytic differentiation of many of the implanted progenitors **Conclusion**: Xenografted adult NSCs become oligodendrocytes and astrocytes, but the efficiency of myelination was difficult to assess

**Table 4:** Studies investigating cell replacement in MS with OPCs

Study	Type of cells	Experimental model	Route of administration	Detection method	Result
Totoiu et al. 2004	Glial-committed progenitor cells	JHMV-infected mice	Injection at T8 spinal cord	BrdU labeling	**Structural:** Transplanted cells survived, colonized long lengths of the spinal cord (up to 12 mm from the implantation site), differentiated into mature oligodendrocytes and remyelinated extensively (up to 67% of axons) Transplanted-remyelinated animals contained approximately 2 × the number of axons within sampled regions of the ventral and lateral columns as compared to non-transplanted animals, suggesting that remyelination is correlated with axonal sparing **Functional:** Locomotor improvement
Wang et al. 2011	Rat OPCs (CG-4)	Zymosan induced focal demyelination model in the spinal cord of rats	Injection in the dorsal column adjacent to the focal demyelination site	GFP-labeled OPCs	**Structural:** Cell replacement: OPCs migrated preferentially toward the inflammatory lesion, survived inside the lesion and a proportion differentiated into mature oligodendrocytes and extensively remyelinated axons within the lesion (colocalization of MBP and GFP)
Franklin et al. 1996	Rat OPCs (CG-4)	EB-X model in rats	Transplantation into the ventral white matter	LacZ labeling	**Structural**: OPCs did not survive in normal tissue. However, they survived in the tissue with lesions Cell replacement: OPCs migrated away from their point of introduction and were able to enter areas of demyelination, and at least some of them differentiate into myelin-forming oligodendrocytes that remyelinate the demyelinated axons therein OPCs transplanted remotely (2-5 mm) from areas of demyelination were unable to migrate through normal white matter **Conclusion**: Transplanted OPCs could not migrate within the normal tissue that separates areas of demyelination. In this case, OPCs transplantation is likely to be successful only if cells are injected either directly into or into the close vicinity of a lesion
Franklin et al. 1995	Rat OPCs (CG-4)	EB-X model in rats	Injection into the lesions	LacZ labeling	**Structural**: Cell replacement: 21 days after transplantation both myelin-forming oligodendrocytes and GFAP(+) astrocytes were identified within the lesion, indicating that the OPCs has bipotential differentiation properties In some areas of the lesion, remyelination was observed that was similar in extent to that achieved by growth factor-expanded populations of O-2A progenitor cells
Tontsch et al. 1994	Rat OPCs (CG-4)	Spinal cord of new-born myelin-deficient rats: PLP mutation	Injection into the spinal cord	LacZ labeling	**Structural**: Cell replacement: in myelin-deficient rat spinal cord, OPCs migrated extensively (up to 12 mm) along the dorsal columns, where they divided and myelinated numerous axons 2 weeks after grafting. Newly formed myelin had a normal ultrastructure and the majority of glial cells appeared to be normal and metabolically active oligodendrocytes with prominent rough endoplasmic reticulum and ribosomes. None of these cells had distended endoplasmic reticulum, the hallmark of md rat oligodendrocytes
Foote et al. 2005	Rat OPCs	Taiep rat (progressive myelin loss): model of chronic demyelination	Injection into the dorsal funiculus of the spinal cord	GFP or LacZ labeling	**Structural:** Myelination competent OPCs could repopulate OPC-depleted chronically demyelinated astrocytosed tissue (but not OPC-containing areas of chronic demyelination), but this repopulation did not result in remyelination Induction of acute inflammation in this non-remyelinating situation could lead to remyelination
Windrem et al. 2004	Fetal and adult human OPCs: A2B5(+) PSA-NCAM(-)	New-born shiverer mice	Injection into the corpus callosum	Immunostaining for human nuclear antigen and MBP	**Structural**: Cell replacement: both fetal and adult OPCs engrafted and differentiated into myelinating oligodendrocytes that mediated the extensive and robust myelination of congenitally dysmyelinated host brain Differences: 1. Fetal OPCs were highly migratory, whereas adult OPCs migrated over shorter distances 2. Adult OPCs gave rise to oligodendrocytes in much higher proportions than their fetal counterparts No adult OPCs became astrocytes in the recipient white matter, whereas 12.7% of fetal OPCs did so by 12 weeks = > adult OPCs behave in a more restricted manner, whereas fetal OPCs acted as glial progenitors 3. Adult human OPCs myelinated shiverer mouse brain much more rapidly (by 6 weeks) than their fetal counterparts (by 12–16 weeks) 4. Adult-derived OPCs matured to ensheath more axons per donor cell than their fetal counterparts
Archer et al. 1997	Mixed glial cell preparation of the oligodendrocyte lineage	Neonatal and adult canine myelin mutant	Injection into two sites of the spinal cord: T13, L2 (within the middle of the dorsal column)	None	**Structural**: Cell replacement: migration and large scale myelination by the transplanted cells, along with long-term survival of these cells and their myelin sheaths Cells at the earlier stages of the oligodendrocyte lineage had the greatest capacity for remyelination following transplantation **Functional**: No evidence of functional improvement
Blakemore et al. 2000	Rat OPCs (mixed glial cell cultures)	Rats with focal demyelination with (1) GalC antibody + complement or (2) lysolecithin, followed by X-irradiation up to the injection point	Injection into 1. the lesion or 2. the dorsal funiculus	None	**Structural**: Cell replacement: in transplanted animals there was more extensive colonization of the demyelination area, as indicated by the presence of remyelination in areas that were not remyelinated in the non-transplanted animals). Transplanted cells could migrate up to 9 mm in 2 months compared to 4 mm recorded for endogenous cells
Sim et al. 2011	Fetal human OPCs (CD140a+) – A2B5+/PSA-NCAM-glial progenitor cells	Shiverer mice	Intracallosal injections	Immunostaining for human nuclear antigen and MBP	**Structural**: Cell replacement: transplanted CD140a(+) cells were highly migratory, differentiated into oligodendrocytes and robustly myelinated the hypomyelinated shiverer mouse brain more rapidly and efficiently than fetal A2B5(+)/PSA-NCAM − cells CD140a+ cells remained bipotential in vivo: they reliably generated oligodendrocytes as well as astrocytes (in a much smaller fraction) in recipient brains CD140a+ cells exhibited both a higher efficiency of oligodendrocytic differentiation, and a more rapid initiation of myelinogenesis, than did fetal A2B5+ OPCs CD140a+ cells myelinated with an efficiency and time course more similar to adult brain-derived A2B5+ OPCs Unlike adult OPCs, however, fetal CD140a+ cells migrated widely, extending as broadly throughout the brain and brainstem as their fetal A2B5+/PSA-NCAM − counterparts, and with even greater penetration of both cortical and subcortical gray matter
Windrem et al. 2008	Fetal human OPCs	Neonatal shiverer mice	Concurrent bilateral hemispheric and cerebellar cell injections	Immunostaining for human nuclear antigen and MBP	**Structural:** Cell replacement: widespread donor cell engraftment throughout the CNS was observed, with robust, efficient and functional host myelination in multiple regions and acquisition of normal nodes of Ranvier and transcallosal conduction velocities, ultrastructurally normal and complete myelination of most axons. Virtually complete chimerization of the murine hosts’ CNSs **Functional**: Prolonged survival and resolution of the neurological deficits
Learish et al. 1999	Rat OPCs	Myelin-deficient rats	ICV	LacZ labeling	**Structural:** Cell replacement: donor cells migrated into the white and gray matter, differentiated into oligodendrocytes and generated normal PLP+ myelin at widespread sites Long-term survival of the transplanted cells Some cells also retained the capacity for astrocytic differentiation Many of the transplanted cells seen in gray matter remained as undifferentiated cells that did not label with known glial markers
Lachapelle et al. 1983	Solid fragments of olfactory bulb from new-born normal mice	Shiverer mice	Intracerebral injection into the rostral thalamus	Anti-MBP No marker for oligodendrocytes	**Structural**: Cell replacement: during a 30–120-day period after implantation: OPCs survived, migrated over long distances, differentiated into oligodendrocytes and produced normal MBP(+) myelin that ensheathed host axons from the level of the graft (rostral thalamus) up to the caudal brain (diencephalon, cerebellum, pons)
Groves et al. 1993	Rat OPCs	EB-X model in rats	Injection into spinal lesions	LacZ labeling	**Structural**: Extensive remyelination was observed after transplantation of OPCs which gives rise to beta-galactosidase-positive oligodendrocytes and remyelinated the demyelinated axons within the lesion
Zhang et al. 1999	Rat OPCs	Myelin-deficient rats	Injection into the spinal cord	Immunostaining for PLP and MBP	**Structural:** Cell replacement: OPCs produced robust myelin after transplantation. 12–14 days after transplantation, a white streak, of average 4 mm length, was present in the dorsal column of the spinal cord of the myelin-deficient rats (which is otherwise semitranslucent because of the lack of myelin). The majority of axons in the dorsal funiculus were myelinated, as confirmed by Toluidine blue-stained semithin sections. The myelin sheaths formed by the transplanted cells were positive for PLP as well as for MBP

#### EAE model

Eight experimental NPCs transplantation studies were performed in the EAE model. All were implemented in rodents, except for the study by Pluchino Gritti et al*.* [62], which was performed in non-human primates and used human-derived NSCs. McIntyre et al*.* in their study used both human and mouse NSCs in discrete experiments, while the remaining studies used rodent-derived NSCs[51, 59, 63–66]. Of the 7 studies using rodent NSCs, in 5 studies, cell replacement was observed, whereas no cell replacement was observed after administration of human NSCs [62, 67].

In the experiment by McIntyre et al*.* (2020), remyelination was observed after transplantation of both human and mouse NSCs intraspinal, but without significant clinical improvement. The beneficial structural effect in the case of mouse NSCs was attributed to cell replacement, whereas in the case of human NSCs to immunomodulation and promotion of endogenous repair. Similarly, in the study by Pluchino Gritti et al*.* (2009), immune regulation, rather than neural differentiation, is proposed as the main mechanism by which human NPCs improve EAE in vivo when the cells were injected IV and ITH. Immune functions, rather than cell replacement, were also attributed to the reduction of demyelination upon transplantation of congenic NPCs into mice with EAE in the experiment of Pluchino et al*.* (2005). IV administered NPCs selectively reached inflamed perivascular regions of the CNS (via activated integrins and chemokine receptors), where they survived as undifferentiated cells and exerted neuroprotective effects, inducing apoptosis of cerebellar T cells and thus protecting against chronic loss of neural tissue. Similarly, in the study by Merzaban et al*.* (2015), after IV injection of NPCs, despite the mitigation of the clinical process, no evidence of long-term stem cell integration was observed and neural repair was attributed to endogenous repair rather than direct cell replacement. Cell replacement was observed in the rest of the studies performed on the EAE model. In the experiment of Ben-Hur et al*.* (2003) injection of rat NPCs ICV or ITH resulted in extensive cell migration exclusively to the infiltrating white matter and expression of astrocytic (GFAP) and oligodendrocyte (NG2, GalC) markers. Another study found that neurospheres administered IV or intra-abdominally reduce demyelination and axonal loss in EAE with a dual mechanism of action, leading to both cell replacement and stimulation of endogenous remyelination [65]. Besides, Giannakopoulou et al*.* (2011) proposed that the stage of the disease has an important role, as the administration of mouse NPCs with bilateral ICV injection, had a different effect in the acute and chronic phase of EAE, with cell replacement occurring only in the chronic phase. The study by Sher et al*.* (2012) used unmodified mouse NSCs, but also NSCs with transient overexpression of the transcription factor Olig2, which is crucial for OPC maturation in myelinating oligodendrocytes [68]. Both cell types injected ICV migrated directly to active lesions, however, only Olig2-NSCs differentiated into OPCs (NG2+), while NSCs remained undifferentiated, expressing nestin [66].

#### Viral demyelination

When NPCs were administered with intracerebral or intraspinal injections to mice with virally induced demyelination after JHMV infection, cell replacement was observed as the transplanted cells migrated to the lesion sites and differentiated into myelinating cells that remyelinated the axons [69, 70]. Furthermore, it was shown that the Olig1 function is essential for the ability to remyelinate direct transplanted NPCs [71]. The Olig1 gene encodes the homonymous transcription factor, which is particularly involved in the development as well as the maturation of oligodendrocytes [72]. Olig1+/+NPCs differentiated mainly into NG2+ OPCs and formed MBP-expressing appendages surrounding the axons, whereas Olig1-/- NPCs differentiated into GFAP+ cells of the astrocytic lineage [71]. Migration into the lesion, differentiation into oligodendrocytes, and extensive remyelination after OPCs administration was also observed in another study with viral demyelination from JHMV [73], in which transplanted-retransplanted animals with OPCs injected at T8 spinal cord, contained approximately twice as many axons as non-transplanted animals, suggesting that remyelination is associated with axonal rescue.

#### Chemical demyelination

Three studies were found using rats with EB-X model. The study by Akiyama et al*.* (2001) used human NPCs, while the other two studies used rat NPCs [74, 75]. The myelinating cells displayed morphological and phenotypic characteristics of Schwann cells [76]. In the study of Keirstead et al. (1999), in which NPCs were directly injected into the lesion, the majority of axons were remyelinated from oligodendrocytes and only 19% were remyelinated from Schwann cells. In the study of Mothe & Tator (2008), transplanted NPCs in the spinal cord differentiated predominantly in an oligodendrocyte direction. However, only some of the oligodendrocyte progeny expressed MBP and remyelinated host axons, while most differentiated into non-myelinating oligodendrocytes. It is, therefore, concluded that NPCs have the intrinsic plasticity to differentiate into oligodendrocytes or Schwann-type cells depending on the host environment, with both cell types capable of myelinating axons [75]. In the experiments of Copray et al*.* (2006) performed with stereotactic implantation of mouse NPCs in the striatum, just below the demyelinated corpus callosum, mice with cuprizone-induced demyelination of the corpus callosum were used as experimental models, which were injected with mouse NPCs, as Olig2-NSCs, in contrast to unmodified NSCs, developed into active myelinating oligodendrocytes that contributed to remyelination [77]. In the study by Einstein et al*.* (2009), after intraventricular injection of NPCs, the observed corpus callosum remyelination was not due to cell replacement, as the transplanted cells did not migrate to the corpus callosum, but remained mostly in the periventricular region in an undifferentiated state. Remyelination was exclusively carried out by the endogenous OPCs of the recipient. Cell replacement was performed in two studies using lysolecithin demyelination rodents as experimental models, which were injected with human NPCs [78, 79]. In the study by Windrem et al*.* (2002), progenitor cells from adult human subcortical white matter were injected into the lesion of dysmyelination, migrated extensively through the demyelination sites and differentiated into myelinating MBP+ oligodendrocytes.

In the experiments of Franklin et al*.* (1995, 1996) and Groves et al*.* (1993), OPCs were administered to adult rats and cell replacement was observed, with OPCs differentiating into myelin-forming oligodendrocytes that remyelinate the stripped axons[80–82]. In the study by Franklin et al*.* (1995), differentiation of OPCs was performed not only in oligodendrocytes but also in GFAP(+) astrocytes within the lesion. Franklin et al*.* (1996) found that administered OPCs do not survive in normal tissue, but survive in tissue irradiated with X-rays or damaged by gliotoxin injection. Transplanted OPCs cannot migrate through normal tissue separating areas of demyelination, leading to the conclusion that transplantation of OPCs is only likely to be successful if cells are injected either directly into or near a lesion [80]. Migration into the lesion, differentiation into oligodendrocytes, and extensive remyelination after OPCs administration was also observed in zymosan demyelination [83].

#### Shiverer model of congenital dysmyelination

Cell replacement occurred in all five studies in which NPCs were transplanted into shiverer mice. In two of them, human NPCs were used [78, 84] while in the others mouse NPCs were used [75, 85, 86]. In all studies, the administered neural progenitor cells migrated extensively, integrated, and differentiated into oligodendrocytes that produced compact functional myelin.

Many of the above studies demonstrate the ability of transplanted human ΟPCs to myelinate the hypomyelinated brain of shiverer animals and improve both their neurological phenotype and lifespan [87–90]. All studies used OPCs of embryonic origin, except for the study by Windrem et al*.* (2004) where adult cells were also used. Both embryonic and adult OPCs were extensively incorporated and differentiated into oligodendrocytes that induced extensive myelination [87–90]. The myelination was so extensive that virtually complete chimerism of the recipient CNS was observed, with mouse gray matter and human-derived white matter glia [89]. However, some differences were observed between cells of embryonic and adult origin. Embryonic OPCs had a high migratory potential, whereas adult OPCs migrated shorter distances. Adult OPCs, however, produced oligodendrocytes in much higher proportions and, unlike embryonic OPCs, produced no astrocytes, indicating that adult OPCs behave in a more restricted manner, while embryonic OPCs act as glial progenitors. Furthermore, adult OPCs myelinated the shiverer mouse brain much faster (6 weeks) than their fetal counterparts (12–16 weeks) and covered more axons per cell [88].

For more efficient isolation of myelinogenic OPCs, Sim et al*.* (2011) selected embryonic human forebrain cells with the marker CD140a, an epitope of platelet-derived growth factor receptor that is specifically expressed by OPCs. Embryonic CD140a+ cells showed in vivo faster and more efficient remyelination, generating both oligodendrocytes and astrocytes (at a much lower fraction) in the recipient’s brains. Furthermore, they were observed to myelinate with an efficiency and time course analogous to adult OPCs, yet migrated widely, extending throughout the brain and stem, like embryonic OPCs. Thus, given their relative homogeneity, ability to migrate widely, and rapid myelinogenesis, CD140a+ cells have a potential advantage as cellular vectors for the treatment of myelin disorders [87, 88]*.*

#### Myelin proteolipid protein (PLP) mutants

Administration of congenic OPCs to myelin-deficient rats with a mutation in the PLP1 gene resulted in cell migration and formation of normal PLP-positive graft-derived myelin [91–93]. In the study by Learish et al*.* (1999), long-term survival of ICV transplanted cells and retention of the capacity for astrocyte differentiation was observed, with a small percentage of astrocytes (< 5%) being donor-derived, although many of the transplanted cells seen in the gray matter remained undifferentiated.

#### Taiep rats (rats with inherited disorder of myelination)

In the experiment of Foote and Blakemore (2005), rat OPCs were administered to the Taiep model via injection into the spinal cord. This study showed that transfected OPCs, as in normal tissue [80] do not enter tissue containing endogenous OPCs, but only in areas where OPCs are depleted. However, even achieving extensive repopulation by OPCs, remyelination was limited to the cell injection site. This study also shows a clear correlation between the induction of acute inflammation and successful remyelination. This adds to the growing body of evidence that acute inflammation provides the stimulus for initiation of remyelination [94] and is evidence for the 'time mismatch’ hypothesis, an explanation for remyelination failure in MS. The absence of an acute inflammatory environment to provide the signals required to promote remyelination will fail the remyelination process [95].

## Cells derived from embryonic stem cells (ESCs)

Embryonic stem cells (ESCs) are pluripotent cells derived from the internal cell mass of the blastocyst and are of interest as candidate cells for the treatment of MS, as they can differentiate into NSCs or OPCs [96]. The ability to form myelin from ESCs has been tested in vivo in a variety of animal models (Table 5). Although NSCs/OPCs derived from ESCs have shown promising preclinical results, their use is constricted due to ethical concerns regarding the source of the cells, as the collection of ESCs destroys the blastocyst. Furthermore, any residual pluripotency from the persistence of some undifferentiated ESCs remains an important safety issue. Thus, alternative sources of stem cells other than internal cell mass are favored for future clinical applications [22].

**Table 5:** Studies investigating cell replacement in MS with ESCs

Study	Type of cells	Experimental model	Administration route	Cell detection method	Result
Brüstleet al. 1999	Mouse ESC-derived glial precursors grown in the presence of FGF2 and PDGF: A2B5(+)	Myelin-deficient rats	Transplantation into the dorsal columns of the spinal cord or ICV transplantation	DNA in situ hybridization with a probe to mouse satellite DNA	**Structural**: Cell replacement: both intraparenchymal and ICV injected ESC–derived glial precursors migrated over several millimeters and differentiated into astrocytes and myelinating oligodendrocytes that generated abundant myelin sheaths. The newly formed myelin sheaths displayed a normal periodicity and variable thickness typical of that seen on remyelination. In contrast to host oligodendroglia, grafted oligodendrocytes found within the myelinated areas had a normal ultrastructural appearance
Glaser et al. 2005	Mouse ESC-derived purified OPCs	Early postnatal (P4–P6) myelin-deficient rats	Injection into the dorsal columns of the spinal cord	DNA in situ hybridization with a labeled probe to mouse satellite DNA and anti-PLP immunolabeling	**Structural**: Cell replacement: 2 weeks after transplantation the engrafted cells had formed PLP(+) myelin internodes/sheaths = > transplanted OPCs differentiated into myelinating oligodendrocytes in vivo [double labeling of PLP-immunolabeled cells with a mouse-specific DNA probe confirmed the donor origin of the engrafted cells.]
Izrael et al. 2007	Human ESCs-derived oligodendrocyte precursors	Shiverer mice	Injection into the third ventricle	Immunostaining for human nuclear antigen and MBP	**Structural**: Cell replacement: human ESCs-NSCs extensively migrated into the brain in vivo and produced numerous long MBP(+) fibers and compact myelin sheaths in different areas after transplantation Pre-treatment by noggin markedly stimulates their capacity to myelinate (highly significant increase in the extent of MBP staining resulting from the noggin pre-treatment of the transplanted cells)
Nistor et al. 2005	Human ESCs-derived OPCs	Shiverer mice	Injected into the dorsal column at T9	BrdU-pre-labeled cells and MBP labeling/immunostaining	**Structural**: Cell replacement: integration of the transplanted cells almost exclusively within white matter, differentiation into mature oligodendrocytes (CC-1+), and multilayered compact MBP(+) functional myelin formation
Zhang PL et al. 2006	Murine ESCs-derived OPCs	Shiverer mice	Transplantation into brain slices	Immunostaining for MBP	**Structural**: Cell replacement: transplanted cells migrated and myelinated axons in the shiverer mouse brain IL6RIL6 acts as an effective stimulator of the myelinating function of ESCs-derived OPCs increasing the extent of remyelinating MBP fibers in the dysmyelinated brain tissue Culture of NSCs with IL6RIL6 chimera enhanced their differentiation into oligodendrocytes with longer and more numerous branches and with peripheral accumulation of MBP in myelin membranes indicating maturation
Aharonowiz et al. 2008	Human ESCs-derived early multipotent NPCs	EAE mice	ICV	Immunofluorescent staining for human-specific mitochondria, human-specific nuclear antigen and GFP labeling	**Structural**: No cell replacement: transplanted NPCs survived and migrated to the host white matter, however, differentiation to mature oligodendrocytes(< 0.01%) and remyelination were negligible Attenuation of the inflammatory process in transplanted animals was observed, which was correlated with the reduction of both axonal damage and demyelination In vitro co-culture experiments showed that hESC-derived NPCs inhibited the activation and proliferation of lymph node-derived T cells in response to nonspecific polyclonal stimuli **Functional**: Transplanted hESC-derived NPCs significantly reduced the clinical signs of EAE **Conclusion**: The therapeutic effect of transplantation was not related to cell replacement for remyelination but was mediated by an immunosuppressive neuroprotective mechanism

### Cell replacement with ESCs

**Fig. 1:**
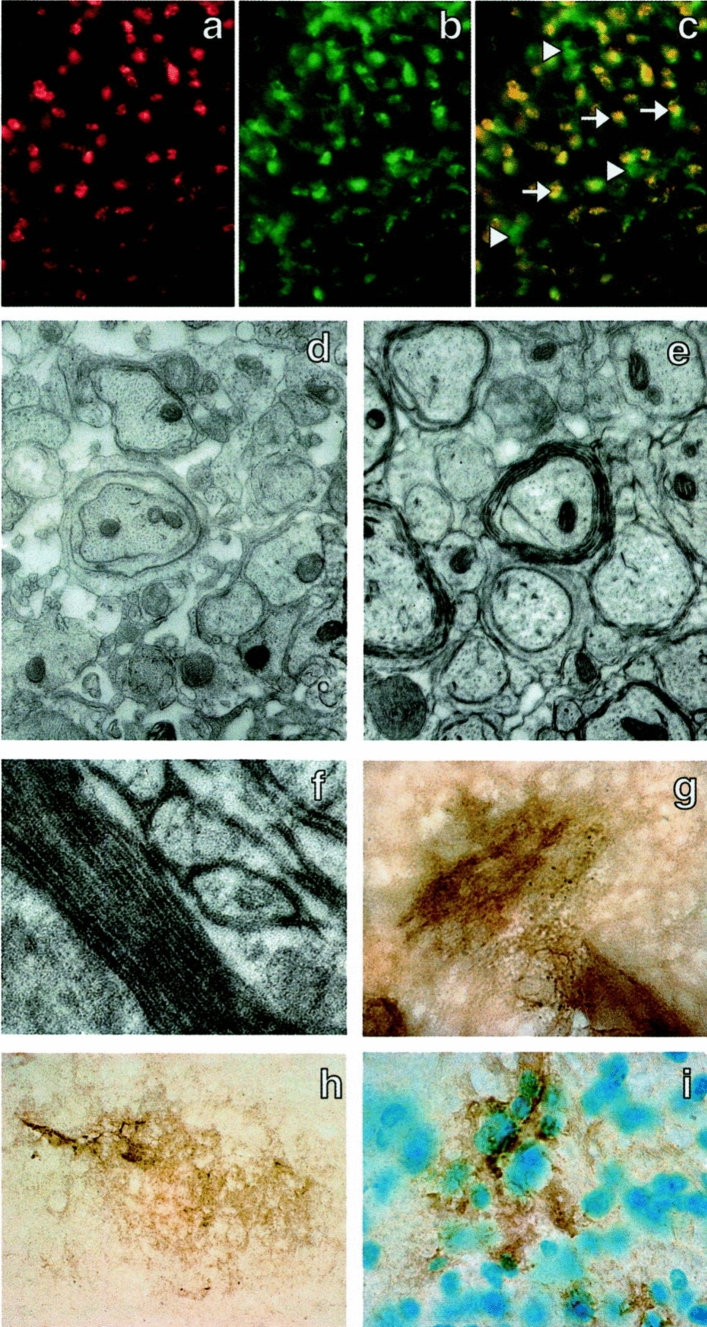
Human embryonic stem cell (hESC)-derived oligodendrocytes integrate, differentiate and display a functional myelinating phenotype following transplantation into the *shiverer* mutant mouse. **A** BrdU immunostaining illustrating the presence of transplanted BrdU pre-labeled cells within the spinal cord white matter. BrdU pre-labeled cells were found almost exclusively within spinal cord white matter. **B** CC-1 immunostaining on the same section as (A) illustrating oligodendrocytes. **C** Composite of BrdU and CC-1 double immunostaining, illustrating that transplanted BrdU^+^ cells adopted the oligodendroglial marker CC-1. Arrows point to double labeled cells, arrowheads point to BrdU-CC-1^+^ cells (endogenous *shiverer* oligodendrocytes). These panels indicate that transplanted BrdU pre-labeled cells survived and integrated within the spinal cord white matter, and became oligodendrocytes. **D** Electron micrograph illustrating that axons of *shiverer* mice are devoid of myelin or are surrounded by one or two uncompacted wraps of myelin. **E** Electron micrograph of the dorsal column white matter of a *shiverer* mouse 6 weeks after transplantation of hESC-derived oligodendrocyte progenitors, illustrating multilayered compact myelin. **F** High-magnification image of compact myelin 6 weeks after transplantation of hESC-derived oligodendrocyte progenitors, illustrating multilayered compact myelin. *Shiverer* mice do not contain multilayered compact myelin. **E, F** Myelination by transplanted cells. **G, H** MBP immunopositive myelin patches within the dorsal column white matter of *shiverer* mice 6 weeks after transplantation of hESC-derived oligodendrocyte progenitors. **I** MBP immunopositive myelin patches within the ventral column white matter of *shiverer* mice 6 weeks after transplantation of hESC-derived oligodendrocyte progenitors; nuclei are in blue. As *shiverer* mice do not produce MBP, G–I demonstrate myelination by transplanted cells. A, B, C, G, H, × 400; D, E, × 20,500; F, × 40,000; I, × 2,000. (Reproduced with permission from Nistor Gi et al*.* 2005 [100])

When administering mouse ESC-OPCs, via ICV or intraspinal infusion, to myelin-deficient rats with dysmyelination due to PLP mutation, they differentiated into oligodendrocytes that myelinated their host axons [97, 98]. Similarly, transplantation of ESCs of both humans [99, 100] and mice [101] in a shiverer model of dysmyelination led to migration and differentiation of cells into mature oligodendrocytes producing MBP+ myelin (Fig. 1).

When administering ESC-derived NSCs to mice with EAE, a significant improvement in the clinical picture was observed with a reduction in the severity of the disease. However, this beneficial effect was not due to cell replacement, but to the immunomodulatory and neuroprotective properties of ESCs [102–104]. In the study of Aharonowiz et al*.* (2008), human ESC-NPCs transplanted within the lateral ventricles of mice with EAE reduced the number of T cells which cause encephalitis, axonal damage, and demyelination. Although NPCs survived and migrated extensively into the brain parenchyma, no differentiation into mature oligodendrocytes occurred, and the extent of remyelination was negligible [102]. In the experiment of Cao et al*.* (2011), mouse ESC-NPCs administered IV in mice with EAE led to a reduction in inflammation and demyelination, but NPCs were rarely detected in the CNS [103]. Furthermore, intra-abdominal administration of human ESC-OPCs in mice with EAE led to a reduction in inflammation and an increase in the number of regulatory T cells within the CNS, while the transplanted cells themselves remained within the ventricular system and did not survive for more than 10 days [104].

Similarly, in the model with demyelination by murine hepatitis virus, human ESC-NPCs that were administered intraductal survived in the parenchyma of the spinal cord for only 1 week, but through immunomodulatory and paracrine effects improved functional effects, reduced demyelination, increased remyelination, and limited neuroinflammation. As the transplanted human NPCs did not differentiate into oligodendroglia, the remyelination appeared in response to the activation of endogenous OPCs via specific factors secreted by human NPCs [105].

## Cells derived from induced pluripotent stem cells (iPSCs)

Reprogrammed cells, having similar characteristics to ESCs, can be differentiated into all cell types, such as NSCs or OPCs (iPSC-NSCs and iPSC-OPCs, respectively). Reprogramming somatic cells of patients into pluripotent cells permit their differentiation in vitro towards desired cell lines or tissues for pathophysiological research or in vivo cell therapy. For the treatment of MS, the differentiation of iPSCs towards oligodendrocyte direction plays a predominant role [106].

However, iPSCs-derived cells cannot yet be used in clinical practice. There is growing evidence that the epigenetic signature of cells can be maintained after induction into iPSCs, leading to issues of immune rejection of grafts [107], or unexpected functions of iPSCs. In one example, iPSC-NSCs grown from patients with PPMS, when transplanted into models of cuprizone-demyelinated mice, exhibited inherent defects, as they lack the neuroprotective phenotype observed in control iPSC-NSCs [108]. Furthermore, the induction process required for the generation of nervous system cells from iPSCs and the expansion to produce enough cells for transplantation is very time-consuming, increasing the likelihood of genetic instability leading to oncogenesis upon transplantation [91].

### Cell replacement with iPSCs

#### iPSC-NSCs

iPSC-NSCs have similar results in animal models of MS, with NSCs from the subventricular zone (Table 6). NSCs from mouse iPSCs, administered intracranially to mice with EAE, provided clinical improvement and reduction of demyelinating areas, axonal damage, and infiltrating inflammatory cells, while no toxicity or tumorigenesis was observed [109]. In addition, they were attracted to damaged areas such as somatic NSCs [65] and were found either in demyelinating lesions or at sites of increased inflammatory cell infiltration. However, most transplanted NSCs did not differentiate in vivo in an oligodendrocyte direction, demonstrating that remyelination is not due to cell replacement. In contrast, iPSC-NSCs, through secretion of a neurotrophin leukemia inhibitory factor (LIF), promoted the survival and differentiation of endogenous OPCs and mature oligodendrocytes [109]. Similarly, a subsequent study observed an improvement in symptoms and reduced T cells via ICV transplantation of iPSC-NSCs at the peak of EAE (18 days after induction) [110].

**Table 6:** Studies investigating cell replacement in MS with iPSCs

Study	Type of cells	Experimental model	Administration route	Cell detection method	Result
Wang et al. 2013	Human iPSC- and ESC-derived OPCs: OLIG2(+) PDGFRα(+) NKX2.2(+) SOX10(+)	Shiverer mice	Bilateral injection into corpus callosum	Immunostaining for MBP and human nuclear antigen	**Structural:** Cell replacement: human iPSC-OPCs migrated widely throughout the shiverer brain, engrafting most densely in white matter, and differentiated as astroglia and myelinogenic oligodendrocytes that robustly myelinated axons, with mature compact myelin and induced nodes of Ranvier The speed and efficiency of myelination by hiPSC OPCs was higher than that previously observed using ESCs-derived OPCs **Functional:** Markedly improved survival
Czepiel et al. 2011	Purified mouse iPSC-derived OPCs	Cuprizone-treated mice with demyelination in the corpus callosum	Injection into the demyelinated corpus callosum	MiniRuby-labeled cells	**Structural:** Cell replacement: over 80% of the implanted cells did not survive the procedure of stereotactic injection and were rapidly removed by microglia. Surviving IPS-derived OPCs of the sorted grafts developed into mature MBP-expressing myelinating oligodendrocytes that contributed to the remyelination of the corpus callosum axons IPS-derived OPCs did not result in teratoma formation, even after a period of over 24 weeks, indicating that no undifferentiated iPSCs were present after the oligodendrocyte differentiation procedure
Najm et al. 2013	Mouse iOPCs	Shiverer mice	Transplantation into the dorsal region of the spinal cord	Immunostaining for MBP	**Structural:** Cell replacement: transplanted iOPCs integrated into the dorsal column white matter of shiverer mice and generated compact MBP+ myelin sheaths around host axons. The myelin was ultrastructurally normal with presence of major dense lines. The g-ratios of myelin produced by 8TF-induced cells was indistinguishable from that of wild-type myelin
Yang et al. 2013	Rat iOPCs (reprogramming from fibroblasts): O4+, NG2+, A2B5+	Neonatal shiverer mice	Bilateral injection into the corpus callosum and cerebellum	Immunostaining for MBP	**Structural: ** Cell replacement: Fibroblast-derived iOPC can differentiate into myelinating oligodendrocytes in vivo. In all 12 injection sites of the 3 grafted brains, small scattered groups of MPB+ cells forming tube-like structures were detected in the cortex, corpus callosum and white matter tract of the cerebellum. Dense myelin sheaths were also observed in the ultrastructural level
Pouya et al. 2011	Human iPSCs-derived OPCs: Olig2+, Sox10+, O4+, A2B5+, NG2+, PDGFRα+	Rat model with focal lysolecithin-induced demyelinated optic chiasm	Injection into the lysolecithin-induced demyelinated optic chiasm (1 week after lysolecithin injection)	DiI-labeling of transplanted cells	**Structural**: Cell replacement: survival, integration of the transplanted cells within the optic chiasm and differentiation into oligodendrocytes that remyelinated effectively. The majority of the grafted cells were PLP+ and/or MBP+, which demonstrated differentiation of human iPSC-derived OPCs into mature oligodendrocytes in vivo and confirmed remyelination by the transplanted cells. A few of the transplanted cells were GFAP+ or MAP2+, which indicated astroglial and neuronal differentiation of the grafted cells in vivo.] **Functional**: Improvement in remyelination of the optic chiasm and optic nerves was functionally evaluated using VEP recording from the skull. It was also shown that hiPSC-derived OPs promoted recovery in transplanted rats compared to vehicle-injected and control rats. This improvement was observed particularly in the first week after cell transplantation, which indicates the short-term effectiveness of this transplantation
Thiruvalluvan et al. 2016	Human iPSC-derived OPCs (5-stage protocol)	1. Cuprizone mouse model 2. EAE mice 3. Marmoset with EAE	1. Injection into the corpus callosum 2. ICV 3. Intracerebral injection (intracortical)	1. Human nucleus immunostaining 2. Luciferase- labeling 3. GFP labeling	**Structural**: 1. Cell replacement: human iPSC-derived OPCs migrated and settled along the axonal fibers of the corpus callosum, and differentiated into mature myelin-forming oligodendrocytes, obtaining a thin, elongated nucleus morphology and expressing MBP 2. No cell replacement: significant reduction of subsequent EAE scores, significant reduction in cell infiltration and decreased demyelination, but no exogenous (grafted) cells could be detected in the lesions. The beneficial effect was most likely caused by secreted protective or anti-inflammatory factors 3. Cell replacement: OPCs selectively migrated toward the MS-like lesions in the corpus callosum and differentiated into MBP(+) myelin-producing oligodendrocytes that myelinated denuded axons. However, no reduction in clinical scores was observed
Douvaras et al. 2014	iPSC-derived OPCs from PPMS patients: O4+	Neonatal shiverer mouse	Injection into the forebrain	Immunostaining for human nuclear antigen	**Structural**: Cell replacement: human iPSC-derived OPCs engrafted into the corpus callosum and differentiated into MBP+ oligodendrocytes that ensheathed host mouse axons. Mature compact myelin with alternating major dense and intraperiod lines was ultrastructurally observed (+ g-ratio measurement = > restoration of normal myelin in several callosal axons) Very few O4-sorted cells underwent differentiation as hGFAP+ astrocytes, that were localized to the subventricular zone and around the ventricles, suggesting that the local environment may induce astrocytic differentiation in these regions
Zhang et al. 2016	Mouse iPSC-derived NSCs: Nestin and Sox2+	EAE mice	Injection into the lateral ventricle	mCherry-labeled iPSC-NSCs	**Structural:** Transplanted iPSC-derived NSCs were integrated into the CNS (in the dentate gyrus) with subsequent dramatic reduction of T cell infiltration and amelioration of white matter damage. iPSC-NSC transplantation promoted remyelination in the marginal zone of the white matter (but not allogenic NSCs) mCherry-RFP was detected to co-localize with the neuron marker Tju1 in the lesion areas showing an integration and differentiation of iPSC-NSC-derived cells into neurons into the damaged tissues **Functional:** Disease symptom score was greatly decreased and motor function was dramatically improved

#### iPSC-OPCs

Another common source of cells for transplantation are OPCs derived from iPSCs. Their in vivo administration in different animal models of MS examined myelin formation from iPSC-OPCs. After their infusion into the cuprizone-induced demyelinated mouse mesenchyme, they differentiated into mature MBP+ oligodendrocytes contributing to the remyelination of neurons [111, 112]. They were also transplanted into, demyelinated from lysolecithin, rat optic chiasm, leading to apparent remyelination. In addition, they were incorporated into the chiasm and differentiated into mature PLP+ and/or MBP+ oligodendrocytes, which remyelinated the axons and contributed to the functional recovery. Some of the transplanted cells also differentiated into GFAP+ astrocytes or MAP2+ neurons [113]. In a genetic model of congenital hypomyelination, administering OPCs derived from human iPSCs to mouse shiverer observed potent derived donor myelination and electron microscopy revealed the production of structurally mature solid myelin, with alternating major dense and intraperipheral lines [114, 115].

Mice transplanted with human iPSC-OPCs improved survival and reduced mortality over a 9-month observation period. In addition, they migrated widely, and differentiated into myelinogenic oligodendrocytes throughout the subcortical white matter and into astrocytes, especially in the central white matter. The speed and efficiency of myelination of human iPSC-OPCs were higher than that of OPCs derived from embryonic tissue [115]. Human iPSC-OPCs generated from MS patients were also injected into shiverer mice and after 16 weeks, human MBP+ oligodendrocytes were detected diffusely throughout the mesocolonium and approximately 30% of the mouse axons were myelinated by them; however, very few differentiated into human GFAP+ astrocytes, localized in the SVZ and periventricular, suggesting that the local environment may induce astrocyte differentiation in these areas [114].

OPCs derived from human iPSCs were also tested in EAE models, where they were transplanted ICV in mice or marmosets with EAE, reducing inflammatory cell infiltration and demyelination, and improving functionality, but without detecting exogenous cells in the lesions. Thus, their beneficial effect did not appear to be due to cell replacement, but most likely to secreted protective or anti-inflammatory factors. However, when transplanted directly into the parenchyma of primates (marmosets) with EAE, the majority of iPSC-OPCs differentiated into mature oligodendrocytes that myelinated the stripped axons, while the remainder retained characteristics of OPCs or differentiated into astrocytes [112].

## Direct transformation of body cells in NSCs and OPCs (iNSCs, iOPCs)

CNS regeneration could also be based on stem cells related to iNSCs and iOPCs that differentiated from somatic cells such as fibroblasts. In this way, they circumvent the problem of multipotency and immunogenicity, and are able to be used in autologous transplantation.

### Cell replacement with iNSCs and iOPCs

#### iNSCs

According to Kim et al*.* (2011) [116], fibroblasts were transformed into iNSCs cells under appropriate culture conditions based on the four Yamanaka reprogramming factors (Table 6). In vitro iNSCs cells grow stably and secrete pro-regenerative molecules such as neurotrophic factors from glial cells (GDNF) and the brain (BDNF). On the other hand, in vivo*,* they can be integrated long-term and functionally into the CNS offering various regenerative applications.

iNSCs cells were transplanted into the cerebellum of 1-day-old shiverer mice, according to dysmyelination models, and differentiated in 10 weeks into functional oligodendrocytes, producing MBP+ myelin [117]. Besides, in demyelinated mouse mesenchyme via cuprizone, transplanted iNSCs differentiated into either oligodendrocytes or astrocytes or remained undifferentiated. Although this transplantation did not reduce demyelination, it succeeded in increasing oligodendrocytes and endogenous OPCs, improving motor deficits. Chronic neuroinflammation and behavioral deficits were ameliorated by transplanted iNSCs cells, which are equivalent to their somatic NSC counterparts and migrate to the meningeal regions of mice, producing proinflammatory MPs. However, few cells proliferated and expressed neuronal, astroglial or oligodendroglial markers [118]. Thus, the therapeutic effect of transplanted iNSCs cells relies not only on cell replacement but also on stimulation of endogenous repair and immunomodulation.

#### iOPCs

According to Najm et al*.* [119], regarding iOPCs cells, fibroblasts through specific transcription factors are directly reprogrammed into myeloid iOPCs without the mediation of iPSCs cells. These surround host neurons, producing structurally compact MBP+ myelin when transplanted into hypomyelinated shiverer mice [119]. Similarly, Yang et al*.* used shiverer mouse brains that were injected with iOPCs cells derived from mouse and rat fibroblasts. These, formed tubular structures around axons at all injection sites expressing myelin, which is present in the CNS, because all MBP+ cells expressed PLP. But they did not produce protein zero, which is the main protein of peripheral myelin. This finding supports the myelinogenicity of iOPCs [120].

## Conclusion

While the treatment of MS continues to evolve, treatment options appear to remain limited in the improvement or prevention of relapses and episodes of acute inflammation in recurrent or active MS. There are no approved interventions capable of effectively promoting the recovery of damaged CNS and stopping the gradual accumulation of disability. Stem cell transplantation is a promising treatment in terms of its regenerative potential, however, most of the preclinical and clinical research has shown that immunomodulatory and trophic properties of stem cells, and not the cell replacement, are the main mechanism of their beneficial effects, making them candidates for the treatment of relapsing or progressive forms of MS. The major hopes for cell replacement and impact on progressive forms of the disease rest on NSCs, somatic or trans-differentiated, for which there is strong preclinical evidence for improving chronic neuroinflammation, but which have not yet been clinically studied to a significant extent in the context of MS [22].

Overall, cell therapies in MS have been experimentally tested for at least 4 decades [121] and significant progress has been made in recent years (Table 7). Hematopoietic and mesenchymal stem cell-based approaches are already in clinical trials. Regarding HSCs, it has strong efficacy in recurrent MS, with markedly better outcomes in patients with active inflammatory disease, short duration of disease, and lower EDSS scores. This is consistent with a therapy that aims to control peripheral immunopathology without directly affecting pathological processes within the CNS. Unfortunately, only one of the studies has shown direct cell replacement when it was injected directly into the striatum and hippocampus and remyelination was performed with different pathways. Transplantation of other types of stem cells, such as MSCs or NSCs/OPCs, may be more useful in patients with progressive forms of MS, where degenerative mechanisms dominate, but this hypothesis has not yet been confirmed [122]. Recent clinical trials are exploiting the immunomodulatory, neuroprotective, and reparative properties of MSCs. Most of the experiments did not show cell replacement despite the positive effect in the reverse of clinical picture and symptomatology which was mainly attributed to the beneficial paracrine action of transplanted MSCs. MSCs administration showed cell replacement only, in one experiment when they were transplanted in the demyelination area and showed no effect in ICV nor IV administration. The use of MSCs has several practical advantages, including relative ease of isolation, mainly when it comes to MSCs of dental pulp or adipose tissue origin, safe administration, and avoidance of the need for immunosuppressive therapy to prevent rejection since autologous transplantation is possible [123]. To date, published clinical trials have been limited to small safety and efficacy studies, and while they have shown a favorable adverse event profile, the efficacy of MSCs transplantation is modest [31].

**Table 7:** Summary table for the mechanisms of action, the advantages, and the disadvantages of the stem cells related to cell replacement in MS

Stem cells	The main mechanisms of action	Pros	Cons
Embryonic stem cells	• Differentiate into neural stem cells or oligodendrocyte progenitor cells • Ability to form myelin • Immunomodulatory and neuroprotective properties	• May potentially serve as an unlimited source of neural cells for transplantation in neurological disorders, such as ms	• Their use is constricted due to ethical concerns as the collection of cells destroys the blastocyst • Any residual pluripotency from the persistence of some undifferentiated embryonic stem cells remains an important safety issue
Induced pluripotent stem cells	• Can be differentiated into all cell types, like neural stem cells or oligodendrocyte progenitor cells • Production of structurally mature solid myelin, with alternating major dense and intraperipheral lines	• Reprogramming somatic cells of patients into pluripotent cells, permits their differentiation in vitro towards desired cell lines or tissues for pathophysiological research or in vivo cell therapy	• Issues of immune rejection of grafts • The induction process is very time-consuming, • Genetic instability leading to oncogenesis upon transplantation
Hematopoietic stem cells	• Replacement of t cell clonotypes especially after autologous hematopoietic stem cell transplantation	• Early treatment has substantial results in improving the clinical picture and prevention of relapses	• All the side effect and risk of autologous hematopoietic stem cell transplantation
Mesenchymal stromal cells	• Selectively migration to sites of tissue damage or inflammation • Enhancement of endogenous repair by stimulation of • Anti-inflammatory activity	• Easily accessible source of autologous or allogeneic somatic stem cells • Escape immunological surveillance • Myelin repair • Suppression of inflammation and immunomodulation • Neuroprotection • Reduced formation of gliotic scar • Promotion of angiogenesis • Direct transfer of mitochondria • Cell fusion	• Formation of cell masses in the brain parenchyma • Focal inflammation • Demyelination • Neuroaxonal loss • Increased collagen and fibronectin deposition
Neural stem cells	• Ability to self-renew and differentiate into functional neurons and glia • Myelinogenic potential • Cell replacement • Immunomodulation • Providing nutritional support, neuroprotection, • Stimulating endogenous remyelination	• Significantly affect the proliferation of the endogenous NSCs • High myelinogenic potential	• Allotransplantation provokes immunological responses to grafted donor cells • Pluripotent stem cells have the potential to generate tumors • The transplantation procedure itself might injure the complicated neuronal circuitry, affecting neurological function
Induced neural stem cells Induced oligodendrocyte progenitor cells	• Cell replacement • Stimulation of endogenous repair • Immunomodulation	• Circumvent the problem of multipotency and immunogenicity, and are able to used in autologous transplantation	• Poor time • Cost efficiency • Potential risk of tumorigenicity • Limited capacity to differentiate into fully functional neurons

NSCs/OPCs transplantation showed a significant positive effect in cell replacement in multiple demyelination models with high percentages of successful outcomes. Only when the injection was IV there was no cell replacement but other ways of remyelination, immunomodulation, and neuroprotection.

While HSCs, MSCs, and NSCs have long been used in preclinical and/or clinical studies for the treatment of demyelination, the successful generation of iPSCs from somatic cells opens a new era in stem cell therapy. The advantages of easy obtaining from the patient’s tissues and good tolerance make stem cells derived from iPSCs or direct trans-differentiation of somatic cells, such as iNSCs and iOPCs, the most suitable candidates for personalized cell replacement therapy. Following trials in EAE and other demyelinating models, stem cell-derived iPSCs have shown significant potential in the treatment of MS when transplanted in the demyelination area. Cell replacement and differentiation into mature oligodendrocytes were observed in most of the experiments when the cell was transplanted directly into the parenchyma. Although there are still many issues to be resolved before clinical application, it is expected that, with the rapid progress in the field of iPSCs, these challenges can be addressed, making iPSC-derived cell transplantation an autologous, safe, and highly effective treatment for MS [22, 106].

While the safety and feasibility of stem cell transplantation have been demonstrated for various cell types and routes of delivery, there is still a need for larger and/or more rigorous studies to quantify the benefits of stem cell therapy and demonstrate superiority over current best treatment models [22]. Thus, all forms of cell therapy for MS should be considered experimental at this time. Rarely, there may be patients with aggressive recurrent MS, with no response to available therapies, for whom autologous HSC transplantation may be warranted. Except for these, cell therapy in MS should only be applied in the context of rigorous clinical trials. In all the cases, comprehensive safety and efficacy data should be collected and reported to existing databases [122].

## Data Availability

All data related to this paper are available upon reasonable request to the first author.
